# Voltaic Narcotism

**Published:** 1859-07

**Authors:** 


*Voltaic Narcotism.*—A paper by Dr. Richardson, was published some time since in the London Medical Times and Gazette, on Voltaic Narcotism. Dr. Waller, Professor of Physiology at the Queen's College, Birmingham, in a subsequent number, indulges in some strictures upon Dr. Richardson's results, and gives an account of a series of experiments of his own upon the same subject.

Without describing his experiments, we give his results in his own words:

"1. Application of a mixture of equal parts of tincture of aconite and chloroform will produce loss of vascularity, and nearly complete anæsthesia after the lapse of ten to fifteen minutes on the human skin.

"2. This anæsthetic is neither retarded nor accelerated by connection with the poles of the voltaic battery.

"3. The anæsthesia produced is circumscribed to the spot on which the narcotizing mixture is applied.

"4. This insensibility is confined to the integuments, and does not extend to the deeper seated parts.

"5. The conclusions of Dr. Richardson relative to painless amputation of the limb of the dog, are not founded on sufficient evidence, as I found that on this animal, amputation of the leg without the use of anæsthetic means, produced exactly the same symptoms as those observed by Dr. Richardson after the use of voltaic narcotism, for upwards of half an hour; the section of the skin and of the tendon of

Achilles, being, according to my experiments on the dog, naturally painless operations.

"6. Dr. Richardson in his experiments on the compression of the sciatic nerve, appears to have overlooked its exact anatomical distribution.

"7. The insensibility is caused by the local absorption of the mixture.

"8. This absorption is in certain cases, sufficient to produce death after the lapse of two hours.

"9. It is not improbable that in infants and children, from the comparative thinness of the cutis, the application of the narcotic over a surface of two or three inches square would be sufficient to produce death.

"10. In all ordinary cases of abraded or cut surfaces, the application of the narcotic mixture may be attended with considerable danger on the adult.

"11. The use of this anæsthetic agent in tooth extraction, is particularly objectionable.

"12. The action of the narcotizing fluid with or without the electric current, when applied to the healthy skin, is attended by a severe local inflammation of a most painful and obstinate character, which would be liable to introduce dangerous complications into surgical operations."

In a subsequent paper, May 14th, Dr. Waller renews his objections, and dwells strongly upon the ill effects of the narcotic mixture He says that if left upon the skin for ten or fifteen minutes it produces vesication, and even destruction of the superficial parts of the cutis, of a very painful and obstinate character. In his own case he found, eight weeks after the application, that the spot was still tender, and unable to localize impressions made upon it. It is darker than the surrounding skin, and devoid of papillæ and hair, while the subcutaneous cellular tissue is thickened.

These are important considerations to those who may feel disposed to try the new process, and should admonish them to enter upon their experiments with the utmost caution.

